# Do higher alarm thresholds for arterial blood pressure lead to less perioperative hypotension? A retrospective, observational cohort study

**DOI:** 10.1007/s10877-022-00889-z

**Published:** 2022-07-07

**Authors:** Agnes S. Meidert, Roman Hornung, Tina Christmann, Elisa Aue, Chetana Dahal, Michael E. Dolch, Josef Briegel

**Affiliations:** 1 Department of Anaesthesiology, University Hospital, LMU Munich, Marchioninistraße 15, 81377 Munich, Germany; 2 Institute for Medical Information Processing, Biometry and Epidemiology, University of Munich, Marchioninistraße 15, 81377 Munich, Germany; 3 Present Address: Helmholtz Zentrum München, Deutsches Forschungszentrum für Gesundheit und Umwelt (GmbH), Independent Research Group Clinical Epidemiology, Ingolstaedter Landstr. 1, 85764 Neuherberg, Germany

**Keywords:** Intraoperative hypotension, Arterial blood pressure monitoring, Alarm threshold, Perioperative monitoring

## Abstract

Arterial blood pressure is one of the vital signs monitored mandatory in anaesthetised patients. Even short episodes of intraoperative hypotension are associated with increased risk for postoperative organ dysfunction such as acute kidney injury and myocardial injury. Since there is little evidence whether higher alarm thresholds in patient monitors can help prevent intraoperative hypotension, we analysed the blood pressure data before (group 1) and after (group 2) the implementation of altered hypotension alarm settings. The study was conducted as a retrospective observational cohort study in a large surgical centre with 32 operating theatres. Alarm thresholds for hypotension alarm for mean arterial pressure (MAP) were altered from 60 (before) to 65 mmHg for invasive measurement and 70 mmHg for noninvasive measurement. Blood pressure data from electronic anaesthesia records of 4222 patients (1982 and 2240 in group 1 and 2, respectively) with 406,623 blood pressure values undergoing noncardiac surgery were included. We analysed (A) the proportion of blood pressure measurements below the threshold among all measurements by quasi-binomial regression and (B) whether at least one blood pressure measurement below the threshold occurred by logistic regression. Hypotension was defined as MAP < 65 mmHg. There was no significant difference in overall proportions of hypotensive episodes for mean arterial pressure before and after the adjustment of alarm settings (mean proportion of values below 65 mmHg were 6.05% in group 1 and 5.99% in group 2). The risk of ever experiencing a hypotensive episode during anaesthesia was significantly lower in group 2 with an odds ratio of 0.84 (p = 0.029). In conclusion, higher alarm thresholds do not generally lead to less hypotensive episodes perioperatively. There was a slight but significant reduction of the occurrence of intraoperative hypotension in the presence of higher thresholds for blood pressure alarms. However, this reduction only seems to be present in patients with very few hypotensive episodes.

## Introduction

During surgical procedures, monitoring and stabilising of vital signs is an essential part of an anaesthetist’s work. Embedded in the monitors software are thresholds which define the safe range of monitored signs. In case of a threshold violation, patient monitors give optical and visual signs, designed to catch the attention of the attending physician. These alarms result in significantly shorter response time of the attending physician: A study by Loeb and colleagues reported a mean response time to abnormal vital signs on the monitor display of 61 s [1], whereas the response time decreased to 1 s for acoustic alarm signals and 6 s for visual alarm signals in a similar study by Morris and Montano [2]. Nowadays, standard monitoring equipment has several different alarms and pre-set thresholds for upper and lower limits of monitored signs. In addition, it is possible to change the limits as appropriate for the individual patient.

One of the key vital signs monitored mandatory in anaesthetised patients is arterial blood pressure. Even short episodes of low intraoperative blood pressure (intraoperative hypotension) are associated with increased risk for postoperative organ dysfunction such as acute kidney injury and myocardial injury [3]. Therefore, timely detection of hypotension is important in order to administer adequate treatment of hypotension. In the past years, a discussion about which blood pressure level is considered safe and does not contribute to postoperative organ dysfunction has been going on [3, 4], resulting in a growing awareness about the role of anaesthetists in avoiding this hazardous condition. Against this backdrop, our institution decided to change the predefined alarm thresholds of the monitoring equipment to tighter limits. Since there is little evidence whether higher alarm thresholds in the patient monitors can help prevent intraoperative hypotension, we analysed the blood pressure data before and after the implementation of altered hypotension alarm settings.

Our hypothesis was that higher alarm thresholds for mean and systolic arterial pressure lead to less hypotensive events during anaesthesia.

## Methods

### Study design and setting

The aim of this retrospective observational cohort study was to investigate the impact of a higher threshold for hypotension alarm at the patient monitor in order to avoid hypotensive episodes during anaesthesia. The local ethics committee (Ethikkommission bei der LMU München, Chairman W. Eisenmenger) approved the study under the protocol number 17-299 on 1st June 2017. The need for informed consent was waived due to the retrospective design.

The study investigated blood pressure values of patients during anaesthesia in a large surgical centre at a German university hospital. The surgical centre contains 32 operating rooms (OR) and 22 induction areas with identical monitoring equipment (M540, Dräger, Lübeck, Germany). Embedded in the monitor software are predefined alarm settings. In July 2016, all patient monitor software was updated. With this update, alarm settings for low arterial pressure had been changed. Previously, the threshold for low mean arterial pressure alarm was set at 60 mmHg, for systolic arterial pressure at 90 mmHg (acoustic and visual alarm). After the update, the monitor alarm went off at 65 mmHg for continuous invasive mean arterial pressure measurement and at 70 mmHg for intermittent oscillometric mean arterial pressure measurement via the upper arm cuff. The threshold for oscillometric systolic arterial pressure was set at 100 mmHg after the update, for continuous measurement it remained at 90 mmHg. Higher thresholds for oscillometric measurements were implemented because of increasing evidence of the method’s tendency to overestimate low blood pressure [5, 6]. All alarm settings could be changed manually as required by the staff.

The anaesthesiologists have not been notified about the change in alarm settings or its reason. We hypothesised that a higher hypotension alarm threshold draws the attention of the attending anaesthesiologist to a decrease in patient’s blood pressure earlier. This would cause earlier treatment and thus reduce the frequency of intraoperative hypotension. To verify this hypothesis we analysed two outcomes: ‘A’, the rate of hypotensive events per patient during anaesthesia before and after the higher hypotension alarm threshold and ‘B’, if the frequency of procedures with no single hypotensive event increased. Intraoperative hypotension is defined as mean arterial pressure below 65 mmHg and systolic arterial pressure below 90 mmHg [7].

### Patients

We included all patients who underwent procedures with an attending anaesthesiologist at the surgical centre in the 3 months before (group 1) and after (group 2) the implementation of altered alarm settings. The time interval was chosen in order to limit the effect of short-term events such as summer holidays. The patients had either general anaesthesia, regional anaesthesia, a combination of both, intravenous analgesia, or monitoring in standby by the anaesthetist. Excluded from the analysis were patients younger than 14 years, cardiac surgery and procedures with extracorporeal circulation or membrane oxygenation. If a patient underwent surgery more than once during the study period, we included only the first procedure.

### Data source and measurements

We extracted systolic and mean arterial blood pressure data from the electronic anaesthesia records (NarkoData®, Imeso, Gießen, Germany) where all vital signs are stored at a sampling rate of 3 min. We screened for invalid measurements (e.g., static pressure during blood withdrawal, failure of the oscillometric measurement) and excluded these values. In addition, duration of anaesthesia, procedure, age, ASA physical status, blood loss and cumulative norepinephrine dose during anaesthesia were analysed based on the electronic anaesthesia record.

### Potential bias

One possible source of bias is that blood pressure during anaesthesia can easily be influenced by the attending anaesthetist who is aware of the study purpose. Since this analysis is a retrospective analysis and staff of the department has not been informed about the change in alarm settings or its purpose this effect does not exist for these data. Another possible source of bias is the summer holiday in the month just after the altered alarm settings. Therefore, we set the study period to 3 months before and after the change of hypotension alarm settings. A sample size calculation was not performed due to the retrospective study design.

### Statistical analysis

Excel (Version 1912, Microsoft, Redmond, Washington) and R (version 4.1, R Core Team, The R Foundation, Vienna, Austria) were used for analysing the data.

Continuous variables were checked visually for normal distribution, where some variables showed strong deviations from normality. Therefore, for all continuous variables the median and the interquartile range (IQR) are presented. Differences between the groups in the 3 months before and after the alteration of alarm settings were analysed using Wilcoxon’s test for quantitative variables and Fisher’s exact test for categorial variables.

### Difference before–after

Intraoperative hypotension is defined as MAP below 65 mmHg and SAP below 90 mmHg [7]. When investigating the effect of changing the alarm thresholds, we considered two outcomes per procedure: (1) the proportion of blood pressure measurements below the threshold among all measurements (outcome A); (2) whether or not at least one blood pressure measurement below the threshold occurred (outcome B). In order to compare the values of outcome A before and after the change of the alarm thresholds, we used quasi-binomial regression adjusting for number of measurements, age, ASA physical status and type of anaesthesia [8]. In the case of outcome B we used logistic regression, again adjusting for number of measurements, age, ASA physical status and type of anaesthesia. Age was included as a potential confounder because the summer holiday fell into the time period after the change of the alarm thresholds. In the summer holiday, traditionally younger patients get their elective surgery. Younger patients tend to be healthier than older patients, which is why less hypotensive events might occur in procedures performed, potentially biasing the comparison between the two groups. The number of measurements was included as a potential confounder to account for the possibility that the durations of the procedures differed between the two groups. The duration has a natural positive influence on the probability of at least one measurement falling below the threshold (outcome B). Moreover, it might exert some influence on the proportion of blood pressure measurements below the threshold among all measurements (outcome A). ASA physical status was included as confounder because hemodynamic instability is more common in patients with relevant comorbidities. In addition, the type of anaesthesia (general anaesthesia, regional anaesthesia, the combination of both or sedation only) can influence a patients’ blood pressure during the procedure. We also investigated whether there was a change in frequency of hypotensive events over time using spline-based quasi-binomial regression and moving averages. Quantile–quantile plots were used to compare the distributions of the values of outcome A before and after adjusting the alarm thresholds. Since the sampling rate of blood pressure values in the electronic anaesthesia records is 3 min, it is not possible to obtain exactly, how long the duration of a hypotensive episode is for each blood pressure value (between 1 and 5 min). We therefore calculated the frequency and proportion of hypotensive measurements rather than the time-weighted average per patient.

### Subgroup analysis

In order to assess whether patients at risk for intraoperative hypotension have fewer hypotensive episodes with higher alarm thresholds, in addition to the main analysis that included all patients, we analysed patients with ASA status III or IV, patients older than 70 years and patients with general or combined general and regional anaesthesia separately. There were 8 subgroups: (A) patients with general anaesthesia, (B) patients with combined general and regional anaesthesia, (C) patients of ASA status 3 or 4, (D) patients older than 70 years, (E) patients older than 70 years and general anaesthesia, (F) patients older than 70 years and combined general and regional anaesthesia, (G) patients of ASA status 3 or 4 and general anaesthesia, (H) patients of ASA status 3 or 4 and combined general and regional anaesthesia. In each subgroup analysis we adjusted for number of measurements and age and the eight P values obtained for the different subgroup analyses were adjusted for multiple testing using the Bonferroni-Holm procedure [9].

## Results

**Fig. 1:**
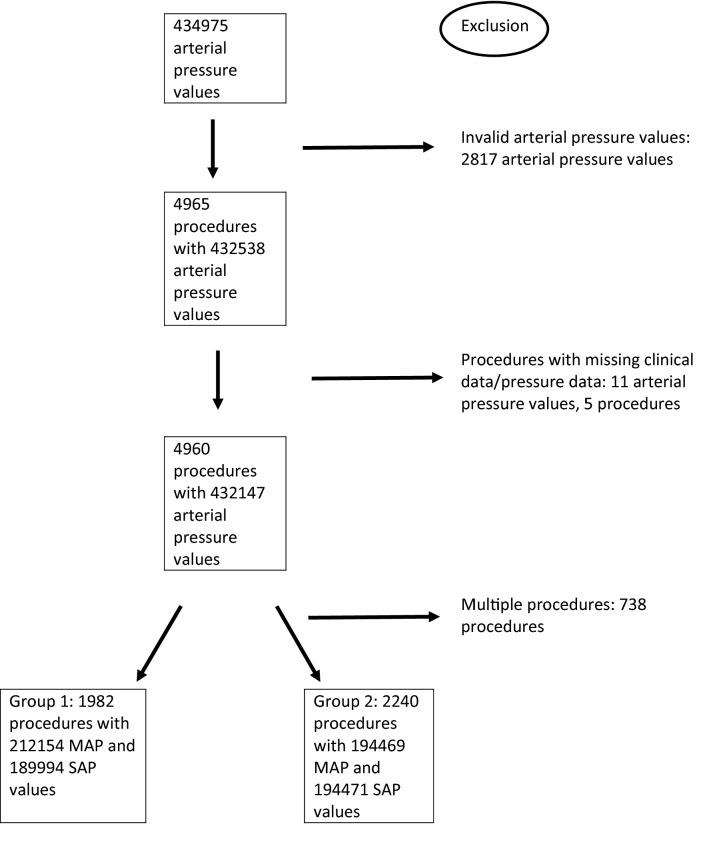
Flow chart of data inclusion

**Fig. 2:**
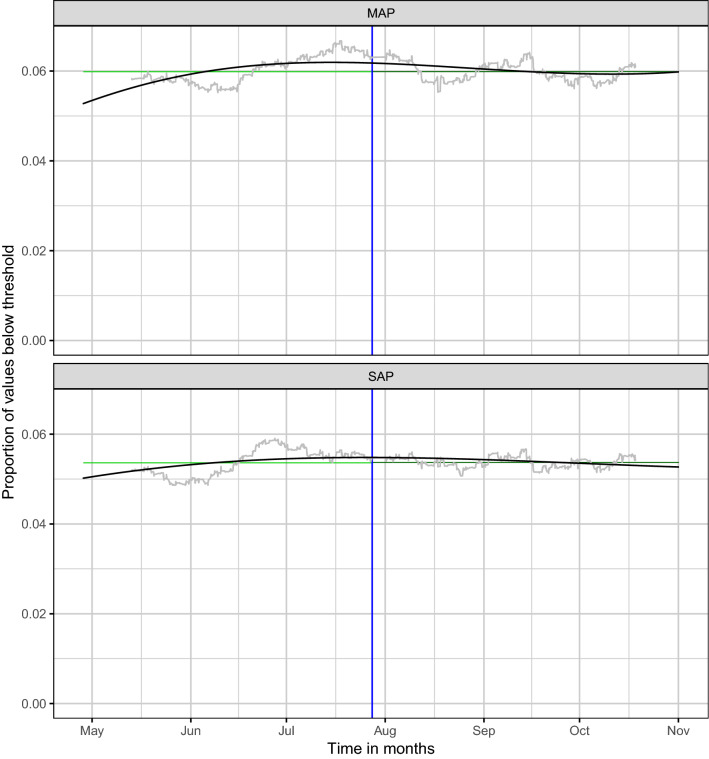
Proportions of values smaller than or equal to the thresholds as a function of time for mean arterial pressure (MAP) in the upper panel and systolic arterial pressure (SAP) in the lower panel. The grey curves show moving averages with window size 800, that is, each value of the curve shows the mean of the 400 proportions left and the 400 proportions right to the respective time point shown on the x axis. The black curves are the fits of quasi-binomial regressions of the proportions on time using B-splines with a cubic spline basis with three degrees of freedom. The blue vertical lines show the time point of implementation of altered alarm settings. The light green and dark green horizontal lines show the means of the proportions before and after the time point of altered alarm settings, respectively

**Fig. 3:**
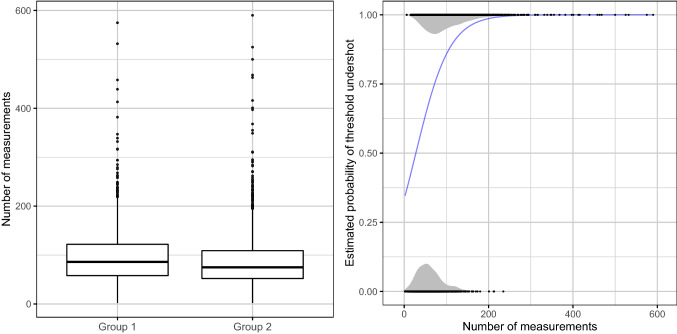
Influence of number of measurements on probability of threshold undershot. Left panel: Numbers of measurements shown separately for each group. Right panel: Estimated probability of no measurement falling below the threshold as a function of the number of measurements. The blue curve shows these probabilities, which were estimated using logistic regression. The black points show the individual numbers of measurements for the cases for which the threshold was not undershot (lower points) and for the cases for which at least one measurement fell below the threshold (upper points). The grey areas show kernel density estimates of the distributions of the black points

After screening for invalid pressure measurements and missing data, a total number 432,158 measurements for systolic arterial pressure and 432,147 for mean arterial pressure were available for analysis. In total there were 4222 patients treated in the observed time frame, where about 9% of these patients were treated several times. The statistical tests we used for testing differences between the two groups assume independency between the observations, which is why it was required that there is only one procedure per patient. Therefore, we excluded multiple procedures per patient by including only the first procedure in cases of patients with several procedures. This left 1982 patients and procedures in group 1 before the altered alarm settings and 2240 patients in group 2 after the altered alarm settings. Detailed data inclusion is presented in Fig. 1. Patients’ characteristics are presented in Table 1. In group 2 there were more patients of ASA physical status 1, cumulative norepinephrine administration and blood loss were statistically significantly lower in group 2, and procedures were on average 16 min shorter. Figure 2 displays the frequency of hypotensive measurements over time, whereas the influence of duration on the probability of experiencing a hypotensive event is shown in the right panel of Fig. 3.

**Table 1:** Patient characteristics

	Total	Group 1	Group 2	p value
**Clinical data**				
Patients, n	4222	1982 (47%)	2240 (53%)	
Age, y	59 (44–72)	59 (45–71)	60 (44–73)	0.384
ASA				0.007
1	456 (10.8)	179 (9.0)	277 (12.4)	
2	1507 (35.7)	711 (35.9)	796 (35.5)	
3	1976 (46.8)	962 (48.5)	1014 (45.3)	
4	274 (6.5)	126 (6.4)	148 (6.6)	
5	8 (0.2)	3 (0.2)	5 (0.2)	
6	1 (0.0)	1 (0.1)	0 (0.0)	
Norepinephrine (mg)	0.32 (0.06–0.97)	0.37 (0.08–1.09)	0.28 (0.06–0.89)	0.001
Total blood loss (ml)	50 (0–300)	100 (0–300)	50 (0–300)	0.004
Duration (min)	160 (101–240)	168 (108–256)	152 (95–227)	< 0.001
**Operation type**				0.109
Trauma/Orthopaedic	840 (19.9)	392 (19.8)	448 (20.0)	
Abdominal	649 (15.4)	312 (15.7)	337 (15.0)	
Ear-nose-throat	580 (13.7)	280 (14.1)	300 (13.4)	
Urologic	521 (12.3)	224 (11.3)	297 (13.3)	
Neurologic	380 (9.0)	182 (9.2)	198 (8.8)	
Vascular	206 (4.9)	91 (4.6)	115 (5.1)	
Gynecologic	204 (4.8)	101 (5.1)	103 (4.6)	
Thoracic	116 (2.7)	63 (3.2)	53 (2.4)	
Skin	138 (3.3)	71 (3.6)	67 (3.0)	
Breast	58 (1.4)	37 (1.9)	21 (0.9)	
Others	530 (12.4)	229 (11.6)	301 (13.4)	
**Anesthesia**				0.001
General	3128 (74.1)	1430 (72.1)	1698 (75.8)	
Both	697 (16.5)	369 (18.6)	328 (14.6)	
Regional	168 (4.0)	68 (3.4)	100 (4.5)	
Not specified	229 (5.4)	115 (5.8)	114 (5.1)	

Quantities are either presented as frequency (%) or median (interquartile range); percentages may not sum up to 100 due to rounding

The number of measurements per procedure (median, IQR) was 81 (55–118) in group 1 and 71 (48–104) in group 2 and ranged from 2 to 590 measurements. The median and IQR of systolic, diastolic and mean arterial pressure per group are presented in Table 2.

**Table 2:** Comparison of group 1 before and group 2 after alarm settings were altered

		Group 1	Group 2	Odds Ratio [CI]	p
Mean arterial pressure	mmHg, median (IQR)	84 (75–95)	84 (75–95)		
Systolic arterial pressure	mmHg, median (IQR)	117 (105–131)	116 (105–130)		
Diastolic arterial pressure	mmHg, median (IQR)	64 (56–74)	64 (56–73)		
**Outcome A**					
Mean arterial pressure	Proportion of hypotensive measurements < 65 mmHg	6.05%	5.99%		0.881
Systolic arterial pressure	Proportion of hypotensive measurements < 90 mmHg	5.47%	5.37%		0.749
**Outcome B**					
Mean arterial pressure	Procedures with ≥ 1 hypotensive event (< 65 mmHg)	80.32%	74.46%	0.84 [0.72, 0.98]	0.029
Systolic arterial pressure	Procedures with ≥ 1 hypotensive event (< 90 mmHg)	78.81%	73.79%	0.89 [0.76, 1.04]	0.135

Quasi-binomial regression was used for testing for differences between mean proportions and logistic regression was used for testing for differences in the frequencies of patients with hypotensive events. In the cases of systolic, diastolic and mean arterial pressure no P values are given, because, as described in methods, restricting our attention to systolic and mean arterial pressure, we compared the tendencies of these values to be hypotensive before and after changing the alarm thresholds

Quasi-binominal regression analysis comparing the differences in proportions of hypotensive events below the threshold of 65 mmHg for mean arterial pressure and 90 mmHg for systolic arterial pressure showed no significant differences before and after the adjustment of alarm settings. The average of values below the threshold were 6.05% in group 1 and 5.99% in group 2 for mean arterial pressure (p = 0.881). Likewise, there was no relevant difference for systolic arterial pressure (Table 2). The risk of experiencing a hypotensive episode during anaesthesia was significantly lower in group 2 with an odds ratio of 0.84 [0.72–0.98] (p = 0.0289). For systolic arterial pressure there was no statistically significant difference between the groups in this respect.

Beyond the non-significant differences between the proportions of hypotensive values before and after adjusting the alarm settings, Fig. 2 does also not indicate any other changes in these proportions over the 3 months period after the alarm settings were altered.

**Fig. 4:**
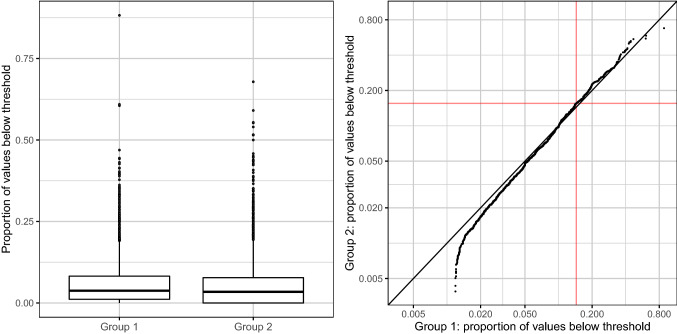
Distributions of proportions of values below hypotension thresholds before and after the adjustment of alarm settings. Left panel: Proportions of values below the threshold separately before and after the adjustment of alarm settings. Right panel: Quantile–quantile plot comparing the distributions of the proportions of values below the threshold before and after the adjustment of alarm settings. In simple terms, the proportions are sorted separately in both groups and subsequently plotted against each other. That is, one point in the plot shows the smallest proportion observed in group 1 plotted against the smallest proportion observed in group 2, another point the second smallest proportion observed in group 1 plotted against the second smallest proportion observed in group 2 and so on. The red lines mark the limits below which 90% of the proportions lie. Zero proportions are not shown because the axes are displayed on a logarithmic scale

The left panel of Fig. 4 shows the distribution of hypotensive values before and after changing the alarm settings. This analysis reveals that hypotensive episodes were less frequent in procedures with a very low proportion of hypotensive events (e.g., patient with transient hemodynamic instability). In contrast, procedures with a very high rate of hypotensive events occurred even more often after the change of alarm thresholds.

The analysis of subgroups A–H did not show any significant differences between the two groups for both outcomes, A and B, after adjusting for multiple testing, for details see Table 3. Of note, in some subgroups the proportions of values in the hypotensive range were higher in group 2 after altering the thresholds (more hypotensive events), but the frequencies of procedures without hypotensive events became higher (less patients ever experiencing hypotension), shown in Table 3. The right panel of Fig. 4 shows a quantile–quantile plot comparing the empirical distributions of the values of outcome A (i.e., the proportions of blood pressure measurements below the threshold among all measurements) before and after changing of the alarm thresholds. This analysis reveals that hypotensive episodes were less frequent only in procedures with a very low proportion of hypotensive events. In contrast, procedures with a very high rate of hypotensive events occurred even more often after the change of alarm thresholds.

**Table 3:** Results of subgroup analysis

MAP	n		Mean proportion < 65 mmHg			Procedures with hypotensive event			
	Group 1	Group2	Group 1 (%)	Group 2 (%)	Adjusted p	Group 1 (n)	Group 2 (n)	Adjusted p	Odds ratio [CI]*
A	1430	1698	6.05	5.80	1	78.95	73.32	0.5997	0.86 [0.72, 1.03]
B	369	328	6.28	7.14	0.9571	89.70	86.28	1	0.78 [0.48, 1.25]
C	1088	1162	6.18	6.12	1	82.81	76.33	0.1876	0.77 [0.62, 0.97]
D	531	672	5.96	6.25	1	80.23	76.49	1	1.00 [0.74, 1.34]
E	344	464	6.19	5.96	1	79.94	76.94	1	1.00 [0.69, 1.43]
F	118	102	5.78	7.07	0.9571	87.29	85.29	1	0.87 [0.39, 1.94]
G	726	787	6.39	5.80	0.9571	82.09	75.35	0.295	0.76 [0.58, 0.99]
H	243	218	6.02	7.28	0.6252	89.71	86.70	1	0.81 [0.45, 1.46]

SAP	n		Mean proportion < 90 mmHg			Procedures with hypotensive event			
	Group 1	Group2	Group 1 (%)	Group 2 (%)	Adjusted p	Group 1 (n)	Group 2 (n)	Adjusted p	Odds Ratio [CI]*
A	1430	1698	5.47	5.22	1	77.41	72.14	1	0.89 [0.75, 1.06]
B	369	328	5.39	5.97	1	87.53	85.37	1	0.91 [0.57, 1.43]
C	1088	1162	5.45	5.44	1	80.51	74.70	0.6042	0.83 [0.67, 1.03]
D	531	672	5.35	5.63	1	78.53	75.45	1	1.04 [0.78, 1.40]
E	344	464	5.54	5.46	1	78.49	75.86	1	1.04 [0.73, 1.49]
F	118	102	4.93	6.24	1	83.05	80.39	1	0.89 [0.44, 1.80]
G	726	787	5.59	5.24	1	79.34	72.68	0.4896	0.78 [0.61, 1.01]
H	243	218	5.2	6.01	1	89.30	86.24	1	0.80 [0.45, 1.45]

*MAP* mean arterial pressure; *SAP* systolic arterial pressure

*Note that the confidence intervals were not adjusted for multiple testing. While the adjusted p values are intended for testing whether the estimated odds ratios significantly differ from 1, the confidence intervals serve the mere descriptive purpose of illustrating the uncertainty in the estimates of the true odds ratios. (A) patients with general anaesthesia, (B) patients with combined general and regional anaesthesia, (C) patients of ASA status 3 or 4, (D) patients older than 70 years, (E) patients older than 70 years and general anaesthesia, (F) patients older than 70 years and combined general and regional anaesthesia, (G) patients of ASA status 3 or 4 and general anaesthesia, (H) patients of ASA status 3 or 4 and combined general and regional anaesthesia

## Discussion

In this study we investigated the impact of altered alarm settings with higher thresholds for low arterial blood pressure on the occurrence of hypotension during anaesthesia in a large retrospective analysis. We found no difference in the mean proportions of hypotensive events when comparing the values of mean and systolic arterial blood pressure of 4222 patients during surgical procedures before and after the adjustment of monitor alarm settings. However, the risk of ever experiencing a hypotensive event with a mean arterial pressure below 65 mmHg was 16% lower in the group monitored with higher pre-set hypotension alarm thresholds. In the subgroup of procedures with a low prevalence of hypotensive events, even less patients were hypotensive after the change of monitor settings. In contrast, for procedures where patients had a high ratio of hypotensive episodes, they experienced even more hypotensive episodes in the presence of higher thresholds than the patients in the 3 months before the altered alarm settings (with lower thresholds). This finding seems to be counter intuitive. One possible explanation is the theory of false alarms. In his essay, Breznitz explains the psychologic phenomenon of ignoring a warning when in the past no negative consequence followed the same alarm [10]. It is possible that anaesthetists disabled the hypotension alarm after noticing that it presumably sounded more often in the time after the altered thresholds. This could have led to more undetected and untreated episodes of hypotension, especially when the alarm sounded frequently throughout the procedure and was subsequently ignored, but this is only speculation. We did not record how thresholds were altered manually by the staff and how often the alarm went off. In a study by de Man, the median alarm frequency was one every 2.9 min with 64% of alarms being irrelevant [11], so one must presume that with higher thresholds, the frequency even increased.

The finding, that the proportion of hypotensive episodes was unchanged in the presence of higher alarm limits and even increased in some subgroups at risk for hypotension (e.g., older than 70 years and combined general and regional anaesthesia) is surprising at first sight. However, our findings are in concordance with the study by Kurz et al. who investigated the impact on triple low alerts [12]. Triple low state is a combination of intraoperative hypotension, deep sedation and low minimum alveolar concentration of volatile anaesthetics, associated with increased mortality in a large retrospective data base analysis [13]. The investigators then designed a study with a real time triple-low alert for the attending anaesthesiologist. Against the expectations, response rates were 51% to the alert and there was no reduction in mortality between the group with alert and controls [12]. This example shows that alarms might be overrated in perioperative medicine. A recent study by Maheshwari and his group investigated the impact of the hypotension prediction index, a software that aims to predict a hypotensive episode, combined with a treatment algorithm. There was no reduction of hypotensive episodes and half of the alarms were ignored by the clinician [14]. In contrast to our finding and the study by Kurz et al., the work by Wijnberge and colleagues evaluating an early warning system for intraoperative hypotension in 60 patients showed a significant reduction of hypotensive episodes in patients monitored with the warning system compared to standard care [15]. The early warning system of Wijnberge’s study was evaluated in a prospective cohort with an observer present and the anaesthesiologist in charge was asked to respond to the alarm of the system (unlike in our study, where the impact of altered alarm settings in a realistic clinical setting was retrospectively analysed). Even in the controlled setting of their study, in 36% of alarms the anaesthetist in charge did not respond to the alarm because of too frequent alarms [15]. So it seems rational at first glance to increase patient safety by setting tight boundaries for a safe range of values and employ alarms in a “better safe than sorry”-approach [16]. However, the numerous alarms sounding in an operating theatre and intensive care unit have been identified to negatively influence both staff and patients [16, 17]. Regarding noise reduction and alarm fatigue, higher alarm thresholds must be considered carefully.

The longer the procedures were in our study, the higher was the rate of threshold undershot. One reason for the higher rate of threshold undershot for longer procedures is certainly that the longer a procedure continues, the more opportunities for undershots arise. However, studies describing the characteristics of intraoperative hypotension by Saugel et al. found, that the lowest blood pressure values occurred typically after the induction before the start of surgery and not so much after hours of surgery [18, 19]. Therefore, the high number of threshold undershots in our study may be partially due to alarm fatigue amongst other factors (e.g. higher blood loss in long procedures).

A potential source of bias of our study was the time of altered hypotension alarm settings shortly before the summer holidays. The patients’ characteristics show that the patients included after changing alarm settings were healthier and had shorter procedures. This may also have contributed to the inability of detecting a reduction in hypotensive rates during surgery, because patients without chronic diseases are less prone to intraoperative hypotension [19]. In addition, shorter procedures had fewer violations of the lower blood pressure limit. When corrected for age, length of the procedure, ASA physical status, and type of anaesthesia, the number of patients monitored with higher alarm thresholds that were never hypotensive was statistically significantly higher. Whether our finding is clinically relevant in terms of less postoperative organ dysfunction remains unclear. The issue needs to be addressed in a larger, prospective multicentre trial. In addition, the question whether tighter alarm limits or better education of anaesthesiologists about the risk of hypotension is more effective in preventing intraoperative hypotension remains unanswered.

Our study has limitations. First, it is a retrospective analysis of a single centre and may therefore not be generalisable to other settings. A prospective, multicentre study should be designed to confirm the findings. Second, it was not possible to analyse the number of hypotension alarms triggered or the manual change of setting by individual physicians.

The strengths of the analysis are the large number of procedures included and that anaesthetists were not informed about the altered settings or its purpose. Therefore, we were able to study the impact of the alarm settings in absence of confounding new institutional protocols.

In conclusion, higher alarm thresholds do not generally lead to less hypotensive episodes perioperatively. There was a slight but significant reduction of the occurrence of intraoperative hypotension in the presence of higher thresholds for blood pressure alarms. However, this reduction only seems to be present in patients with very few hypotensive episodes.

## Data Availability

The dataset used for the study is available from the corresponding author on reasonable request.
